# Ectopic Expression of PAP1 Leads to Anthocyanin Accumulation and Novel Floral Color in Genetically Engineered Goldenrod (*Solidago canadensis* L.)

**DOI:** 10.3389/fpls.2019.01561

**Published:** 2019-11-27

**Authors:** Oded Skaliter, Jasmin Ravid, Elena Shklarman, Nadav Ketrarou, Noam Shpayer, Julius Ben Ari, Gony Dvir, Moran Farhi, Yuling Yue, Alexander Vainstein

**Affiliations:** ^1^The Robert H. Smith Faculty of Agriculture, Food and Environment, Institute of Plant Sciences and Genetics in Agriculture, The Hebrew University of Jerusalem, Rehovot, Israel; ^2^The Laboratory for Mass Spectrometry and Chromatography, The Robert H. Smith Faculty of Agriculture, Food and Environment, The Hebrew University of Jerusalem, Rehovot, Israel

**Keywords:** anthocyanin, color, flavonoid, regeneration, *Solidago*, transformation

## Abstract

Floral pigmentation is of major importance to the ornamental industry, which is constantly searching for cultivars with novel colors. Goldenrod (*Solidago canadensis*) has monochromatic yellow carotenoid-containing flowers that cannot be modified using classical breeding approaches due to a limited gene pool. To generate Solidago with novel colors through metabolic engineering, we first developed a procedure for its regeneration and transformation. Applicability of different cytokinins for adventitious regeneration was examined in the commercial cv. Tara, with zeatin yielding higher efficiency than 6-benzylaminopurine or thidiazuron. A comparison of regeneration of commercial cvs. Tara, Golden Glory and Ivory Glory revealed Tara to be the most potent, with an efficiency of 86% (number of shoots per 100 leaf explants). Agrobacterium-based transformation efficiency was highest for cv. Golden Glory (5 independent transgenic shoots per 100 explants) based on kanamycin selection and the GUS reporter gene. In an attempt to promote anthocyanin biosynthesis, we generated transgenic Solidago expressing snapdragon (*Antirrhinum majus*) Rosea1 and Delila, as well as *Arabidopsis thaliana* PRODUCTION OF ANTHOCYANIN PIGMENT 1 (PAP1) transcription factors. Transgenic cv. Golden Glory expressing cauliflower mosaic virus 35S-driven *PAP1* generated red flowers that accumulated delphinidin and its methylated derivatives, as compared to control yellow flowers in the GUS-expressing plants. The protocol described here allows efficient engineering of *Solidago* for novel coloration and improved agricultural traits.

## Introduction

Fruit and flower colors play a crucial role in plants’ attractiveness to pollinators and seed dispersers (Kevan and Baker, 1983). Pigmentation also plays a major role in the commercial success of most crops on the market. The three main groups of pigments in plants are carotenoids, anthocyanins, and betalains (Grotewold, 2006; Tanaka and Ohmiya, 2008). In some crops, classical breeding approaches have yielded an array of colors; however, others, such as goldenrod (*Solidago canadensis*) and gypsophila (*Gypsophila paniculata*), remain monochromatic due to a limited gene pool. *Solidago* (family Asteraceae) is a perennial plant that can reach up to three waves of flowering per year. The radiate flower heads are comprised of distinct ray and disc florets, and numerous heads are usually grouped in complex inflorescences. It is used mainly as a filler to complement floral arrangements. All the varieties of *Solidago* existing today have carotenoid-derived yellow flowers (Horvath et al., 2010). Carotenoids are organic molecules, usually C40, that are produced by a variety of organisms, including bacteria, fungi, algae, and plants.

Another class of pigments, anthocyanins, are flavonoids that derive from the general phenylpropanoid pathway, the primary precursor of which is the amino acid phenylalanine. Anthocyanins accumulate in the vacuoles of epidermal and subepidermal cells of flowers, fruit, and leaves (Forkmann, 1991). Anthocyanins derive from six main anthocyanidins: pelargonidin, cyanidin, delphinidin, peonidin, petunidin, and malvidin (Liu et al., 2018). Depending on pH, secondary modifications (glycosylation, acylation, and methylation), cofactors, etc., anthocyanins yield orange, red, or blue colors (Yonekura-Sakakibara et al., 2009; Khoo et al., 2017). In many species, flavonoid biosynthesis has been shown to be regulated by the MYB–bHLH–WD40 (MBW) ternary complex (Zhang et al., 2003; Koes et al., 2005; Ramsay and Glover, 2005; Schwinn et al., 2006; Albert et al., 2014; Verweij et al., 2016).

Many studies over the years have used genetic engineering to change flower color. For example, introduction of the biosynthetic enzyme flavonoid 3′,5′-hydroxylase (F3′5′H) to chrysanthemum, rose, and carnation leads to transgenic flowers that accumulate delphinidin and its derivatives (Katsumoto et al., 2007; Brugliera et al., 2013). In addition to biosynthetic enzymes, transcription factors have also been harnessed to generate new colors in plants. *Arabidopsis thaliana* PRODUCTION OF ANTHOCYANIN PIGMENT 1 (PAP1) is a MYB family transcription factor that positively regulates biosynthetic genes in the phenylpropanoid pathway (Borevitz et al., 2000; Mathews et al., 2003; Ben Zvi et al., 2012). In PAP1‐overexpressing petunia flowers accumulating increased levels of anthocyanins, mRNA levels of l‐phenylalanine ammonia lyase (PAL), cinnamic acid‐4‐hydroxylase (C4H), flavanone 3‐hydroxylase (F3H), and dihydroflavonol‐4‐reductase (DFR) were elevated (Ben Zvi et al., 2008). Ectopic expression of *PAP1* in flowers of other species, such as rose, hop, and tobacco, also led to increased levels of anthocyanins (Xie et al., 2006; Ben Zvi et al., 2012; Gatica-Arias et al., 2012). Delila (Del) and Rosea1 (Ros1) are, respectively, bHLH and MYB family transcription factors from snapdragon (*Antirrhinum majus*) that, similar to PAP1, positively regulate anthocyanin-biosynthesis genes. Ectopic expression of these genes resulted in the accumulation of anthocyanins, even in fruit that contained only carotenoids (Butelli et al., 2008; Schwinn et al., 2014; Naing et al., 2017).

In this work, we developed a transformation and regeneration system for *S. canadensis*, with the aim of generating transgenic plants with a new flower phenotype. We established transgenic lines that constitutively express the transcription factor *PAP1* and succeeded in obtaining *Solidago* plants with red-pigmented flowers.

## Materials and Methods

### Plant Material

Sterile prerooted cuttings of *S. canadensis* cvs. Tara (TA), Golden Glory (GG), and Ivory Glory (IG) were obtained from Danziger “Dan” Flower Farm (Moshav Mishmar Hashiva, Israel) and maintained on full-strength MS medium (Murashige and Skoog, 1962) with 3% (w/v) sucrose solidified with 8 g L^−1^ agar (MS30). The pH of the medium was adjusted to 5.8 prior to autoclaving for 20 min at 121°C.

### Shoot Regeneration

Leaf explants (ca. 1 cm) were placed (adaxial side up) on petri dishes with regeneration medium (REM) composed of MS30 supplemented with 0.1 mg L^−1^ α-naphthaleneacetic acid (NAA) and 1.5 mg L^−1^ of one of the following cytokinins: benzyl aminopurine (BA), zeatin, or thidiazuron (TDZ). The explants were cultured in a growth chamber at 25 ± 1°C with 16-h photoperiod under cool white light (60 µmol m^−2^ s^−1^) and subcultured on fresh medium every 10 days. Shoots of about 1 cm in length were transferred to elongation medium (EM) composed of MS30 supplemented with 0.1 mg L^−1^ NAA and 0.5 mg L^−1^ BA. Elongated shoots were transferred to rooting medium composed of MS30 supplemented with 0.1 mg L^−1^ NAA and 0.5 mg L^−1^ gibberellic acid.

### Bacterial Strains and Vectors

*Agrobacterium tumefaciens* strain EHA105 carrying the binary vector pKIWI105 (Janssen and Gardner, 1990; Ben Zvi et al., 2012) was used for transient transformation. For stable transformation of *Solidago*, we used *A. tumefaciens* strain AGLO pCGN7001 (Ben Zvi et al., 2012). The plasmids carried neomycin phosphotransferase II (*nptII*) driven by either a NOS promoter (pKIWI105) or 35S promoter (pCGN7001). pKIWI105 and pCGN7001 carried the *uidA* gene encoding β-glucuronidase (GUS) driven by either an 35S promoter (pKIWI105) or a mannopine synthase promoter (pCGN7001). The GUS-encoding gene is not expressed in agrobacterium cells carrying pKIWI105 due to lack of a bacterial ribosome-binding site, making this plasmid suitable for evaluation of transient transformation (Janssen and Gardner, 1990). *Solidago* PAP1-overexpressing lines were obtained using *A. tumefaciens* strain AGLO carrying 35S:PAP1 in the binary vector pCGN1559 (Ben Zvi et al., 2012). The genomic sequence of *PAP1* (GenBank accession no. AF325123) was amplified from *Arabidopsis* strain Columbia using the following primers: forward 5′-ATCTGCAGACTTATACCTTTTACAATTTGTTTA-3′ and reverse 5′-TCAAACTGCAGAAACTAAGCCCA-3′ and cloned into a pCD intermediate vector between 35S promoter and OCS terminator. This cassette was then cloned into pCGN1559. The binary vector pJAM1890 containing GTW : *Ros1* and 35S:*Del* genes (Butelli et al., 2008) was kindly provided by Prof. Cathie Martin’s laboratory. The GTW promoter used to drive the expression of *Ros1* was replaced with 35S, and this vector was transformed into *A. tumefaciens* strain AGLO. The latter was used in transformation processes to obtain 35S:*Del*/35S:*Ros1*-transgenic *Solidago* lines.

### *Agrobacterium*-Mediated Transformation

Bacterial cells were streaked on petri dishes containing 1.5 g L^−1^ agar-solidified Luria Bertani (LB) medium with the following antibiotics: 50 mg L^−1^ rifampicin and 40 mg L^−1^ gentamycin for selection of pCGN, or 50 mg L^−1^ kanamycin for selection of pJAM1890 and pKIWI105, and incubated in the dark at 28°C for 48 h. Bacteria from a single colony were cultured at 28°C for 20 h in liquid LB medium on a rotary shaker (200 rpm). The medium was supplemented with 100 µM acetosyringone, and the appropriate antibiotics. After reaching OD_550_ = 0.5, the bacteria were harvested and resuspended in liquid cocultivation medium (CCM) composed of MS30 supplemented with 100 µM acetosyringone.

### Optimization of Transient Transformation

Leaf explants were immersed for 10 min in a bacterial suspension of *Agrobacterium* EHA105 carrying pKIWI at OD_550_ = 1. Inoculated leaves were then blotted dry and cultured on solid CCM for a period of up to 3 days. Following cocultivation, leaf explants were histochemically evaluated for transient GUS expression by counting the number of GUS-expressing leaves on explants, as well as the number of blue spots per explant, under a stereomicroscope.

### Transformation and Regeneration of Transgenic Plants

Leaf explants were immersed in a bacterial suspension (OD_550_ = 0.2). After 3 days of culture on CCM, the explants were transferred to REM (1.5 mg L^−1^ zeatin) supplemented with 200 mg L^−1^ carbenicillin and 70 mg L^−1^ kanamycin. After 14 days of culture, the explants were transferred to fresh medium and cleaned of occasionally developing shoots. After 2 additional weeks, clusters of regenerated adventitious shoots were excised from the primary explants and cultured on REM supplemented with 200 mg L^−1^ carbenicillin and 60 mg L^−1^ kanamycin for adventitious shoot regeneration and selection of transgenes. After 12 weeks, new adventitious shoots that emerged from axillary buds were subcultured on EM supplemented with 200 mg L^−1^ carbenicillin and 50 mg L^−1^ kanamycin. Elongated shoots were transferred to rooting medium supplemented with 200 mg L^−1^ carbenicillin and 50 mg L^−1^ kanamycin. After 8 weeks of culture, roots were cleaned of agar and regenerated plants were transferred to soil and grown in a growth room at 23°C under constant light for a hardening period of 4 weeks. The transgenic plants were then transferred to the greenhouse for their further development and flower production.

### Histochemical Assay of GUS Activity

A histochemical assay of GUS activity was performed according to Stomp (1992). Tissue samples were incubated for a few hours to overnight at 37°C in a 0.1% (w/v) X-Gluc (5-bromo-4-chloro-3-indolyl β-D-glucuronic acid sodium salt; Biosynth Inc., Staad, Switzerland) solution containing 0.1 M sodium phosphate buffer (pH 7.5), 10 mM EDTA, and 0.1% (w/v) Triton X-100. When necessary, green tissues were bleached, after staining, by immersion in 50% EtOH for a few hours, followed by several washes with 70% EtOH. It should be noted that no background GUS activity was detectable in any of the analyzed tissues of the control non-transgenic plants.

### RNA Analyses

For RT-PCR analyses, total RNA (10 µg) was isolated as previously described by Ben Zvi et al. (2012) and treated with RNase-free DNase (Promega, Madison, WI). cDNA was generated using oligo(dT)15 primer and M-MLV reverse transcriptase (both from Promega). To confirm that the generated samples were not contaminated with DNA, PCR amplification was also conducted with samples generated without reverse transcriptase. The primers used for the RT-PCR experiments were: GUS forward 5′-TTTAACTATGCCGGGATCCATCGC-3′, reverse 5′-CCAGTCGAGCATCTCTTCAGCGTA-3′; PAP1 forward 5′-ACGCCCATTCCTACAACAC-3′, reverse 5′-TCTCTCCATCGAAAAGACTCC-3′; actin forward 5′-GGTTTTGCTGGGGATGATGC-3′, reverse 5′-CATTGAATGTCTCAAACATGATTTGAGTC-3′; Ros1 forward 5′-TGTGAGAAGCAAACGC-3′, reverse 5′-TTAATTTCCAATTTGTTGGG-3′; Del forward 5′-TGGTC′CAATTCAGTTGCACAA-3′, reverse 5′-CGGCTCCAATCTTGTCCGT-3′; PAL forward 5′-TTGGTGCTACTTCTCANNGGAG-3′, reverse 5′-TTAGGACCTTTTTNGCTACTTGGC-3′; F3H forward 5′-CNGTGCAAGATTGGAGGGAGATTG-3′, reverse 5′-CCNTTGCTCAAATAATGTCCATGATCNCC-3′. The predicted sizes of the amplified DNA fragments were 428, 354, 328, 354, 1554, 986, and 367 bp for GUS, PAP1, actin, Ros1, Del, PAL, and F3H, respectively. GUS, PAP1 and actin (used as a reference for validation of cDNA intactness in all samples) cDNA amplification was conducted with an initial denaturation step of 94°C for 3 min, followed by 30 cycles of 10 s at 94°C, 10 s at 55°C and 20 s at 72°C, and a final elongation step at 72°C for 10 min. Real-time quantitative PCR (qPCR) of PAP1 was performed for 40 cycles (94°C for 15 min and then cycling at 94°C for 10 s, 60°C for 30 s, and 72°C for 20 s) in the presence of Absolute Blue qPCR SYBR Green ROX Mix (Thermo Fisher Scientific, Waltham, MA) on a Corbett Research Rotor‐Gene 6000 cycler. A standard curve was generated using dilutions of cDNA samples, and data analysis was performed using Rotor‐Gene 6000 series software 1.7. Primer specificity was determined by melting‐curve analysis; a single, sharp peak in the melting curve ensured that a single, specific DNA species had been amplified. The primers used were: PAP1 forward 5′-GTCCAAAGGGCTGCGAAAAG-3′, reverse 5′-CGGTTTAGCCCAGCTCTTACA-3′; actin forward 5′-TGCTGATCGTATGAGCAAGGAA-3′, reverse 5′-GGTGGAGCAACAACCTTAATCTTC-3′.

### Experimental Design and Statistical Analysis

For evaluation of transient/stable transformation efficiencies, at least three independent experiments (n = 3) were performed and similar results were obtained with all replicates. Transformation efficiencies, presented as percentage of GUS-expressing explants, were determined using at least 100 stem explants per treatment. Statistical analysis was conducted on means of independent experiments, and data were square-root arcsine-transformed prior to statistical analysis. One-way ANOVA was used, and means were compared using Tukey–Kramer HSD test (*P* ≤ 0.05). All statistical calculations were performed using JMP 5 (JMP software, SAS Institute Inc., Cary, NC).

### Anthocyanin Contents

*Solidago* flowers were collected and immediately frozen in liquid nitrogen. Tissue (100 mg) was ground in a Tissue Lyser (Retsch/Qiagen, Hilden, Germany) and anthocyanins were extracted from flowers with methanol containing 1% (v/v) HCl (100 mg of fresh tissue per 1 ml of acidic methanol). Absorption values (A) of the extract at 530 and 657 nm were measured using the formula A_530_ − 0.25(A_657_), which allows for subtraction of chlorophyll interference. Three biological replicates per treatment were used for analyses of anthocyanin levels.

### LC–MS Analysis

Leaf and flower samples (∼50 mg) were extracted with methanol, assisted by probe sonication. Extracts were dried under a nitrogen stream, redissolved in 1 ml methanol:water (1:1, v/v), and filtered through regenerated cellulose-membrane filters (0.2 µm) before LC–photodiode array–MS analysis.

Samples were analyzed with a LC–MS system consisting of a Dionex Ultimate 3000 RS HPLC coupled to a Q Exactive Plus hybrid FT mass spectrometer equipped with heated electrospray ionization source (Thermo Fisher Scientific). The HPLC system consisted of a quaternary pump, thermostatted autosampler, thermostatted column compartment, and photodiode array detector. Chromatographic separation of the compounds was carried out on a Luna Omega PS C18 column (2.1 × 100 Mm, 1.6 µm, Phenomenex) using a linear gradient of (B) acetonitrile and (A) water with 0.1% (V/V) acetic acid. Separation conditions were as follows: from T = 0 to T = 1 min, B = 10%; T = 12 min, B = 95%; T = 16 min, B = 95%; oostrun equilibrium time 4 min; flow rate 0.35 Ml min^-1^; column temperature 40°C; volume of injection 5 µl. the UV chromatogram was recorded at wavelength λ = 520 nm and UV–Visible Spectrum-Acquisition Range was 230–700 nm. The mass spectrometer was operated in positive and negative electrospray ionization modes (separate runs), and ion source parameters were as follows: spray voltage 3 Kv; capillary temperature 300°C; sheath gas rate (Arb) 40; and auxiliary gas rate (Arb) 10. Mass spectra were acquired in full scan (M/Z 150–800) and all ion-fragmentation (M/Z 80–800) Da acquisition modes at resolving power 70.000. The LC–MS system was controlled and data were analyzed using xcalibur software.

## Results

### Adventitious Regeneration of *Solidago*

**Figure 1:**
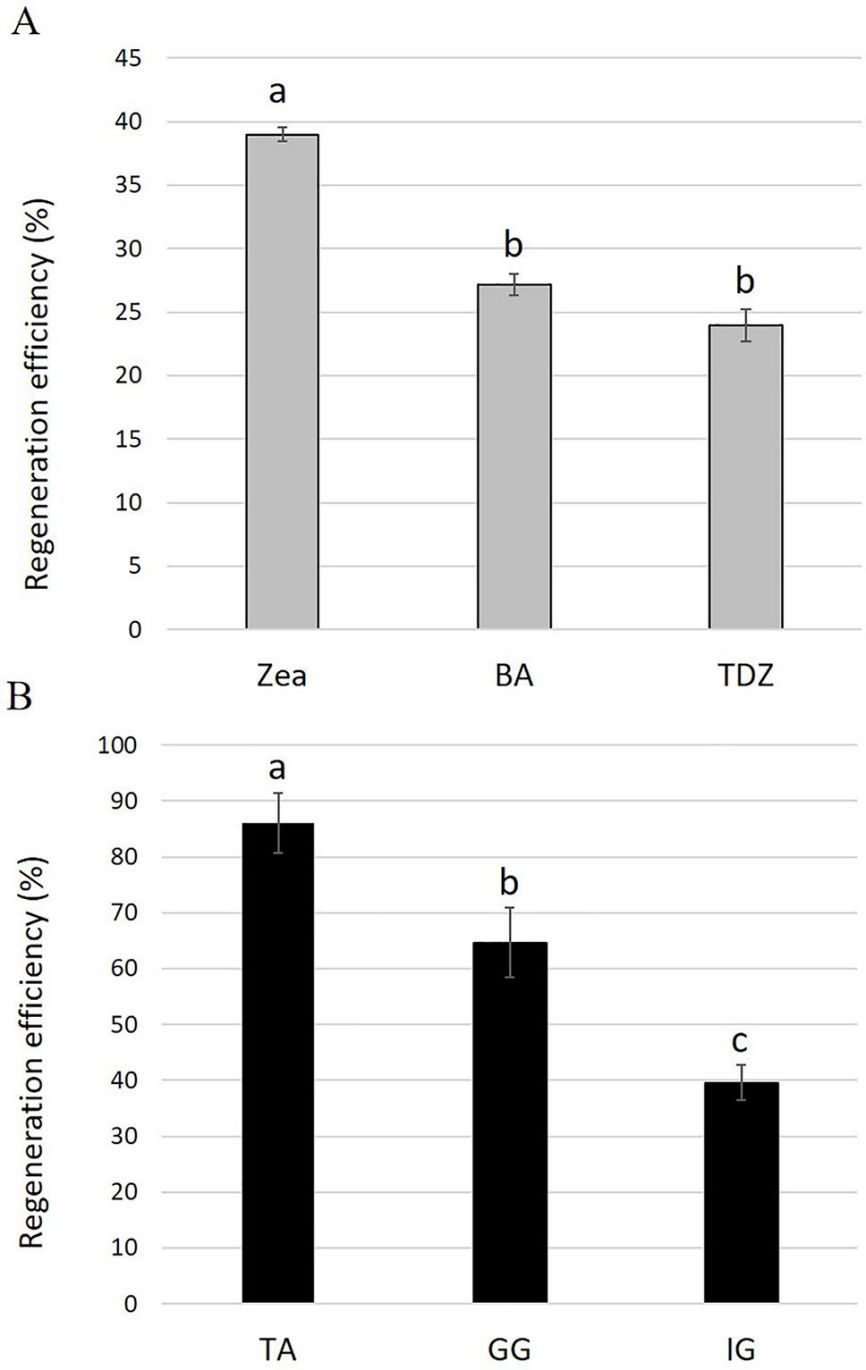
**(A)** Regeneration efficiencies for *Solidago* cv. Ivory Glory leaf explants cultured on media containing different types of cytokinins: zeatin (Zea), benzyl aminopurine (BA), or thidiazuron (TDZ). **(B)** Regeneration efficiencies of different *Solidago* cultivars. Young leaf explants of cvs. Tara (TA), Golden Glory (GG), and Ivory Glory (IG) were grown on zeatin-containing media. Regeneration efficiency was calculated as number of independent shoots developed from 100 leaf explants, presented as %. Bars represent mean values of three independent experiments ± SE. Signiﬁcance of differences between treatments was calculated using Tukey–Kramer HSD test following one-way ANOVA. Values with different lowercase letters are significantly different (*P* ≤ 0.05).

To examine the effect of different cytokinins on adventitious regeneration, young leaf explants derived from 4-week-old tissue-culture-grown plantlets of *S. canadensis* cv. IG were placed on REM containing 0.1 mg L^−1^ NAA supplemented with 1.5 mg L^−1^ BA, zeatin, or TDZ. After 22 days in tissue culture, the number of independent adventitious shoots regenerated from each explant was counted. The highest regeneration efficiency was observed on REM supplemented with zeatin: 34 explants with 1.16 adventitious shoots per explant (out of 100 explants), yielding an overall regeneration efficiency of 39% (**Figure 1A **and **Table S1**). The regeneration efficiencies on REM supplemented with BA and TDZ were lower, 27% and 24%, respectively (**Figure 1A**).

**Figure 2:**
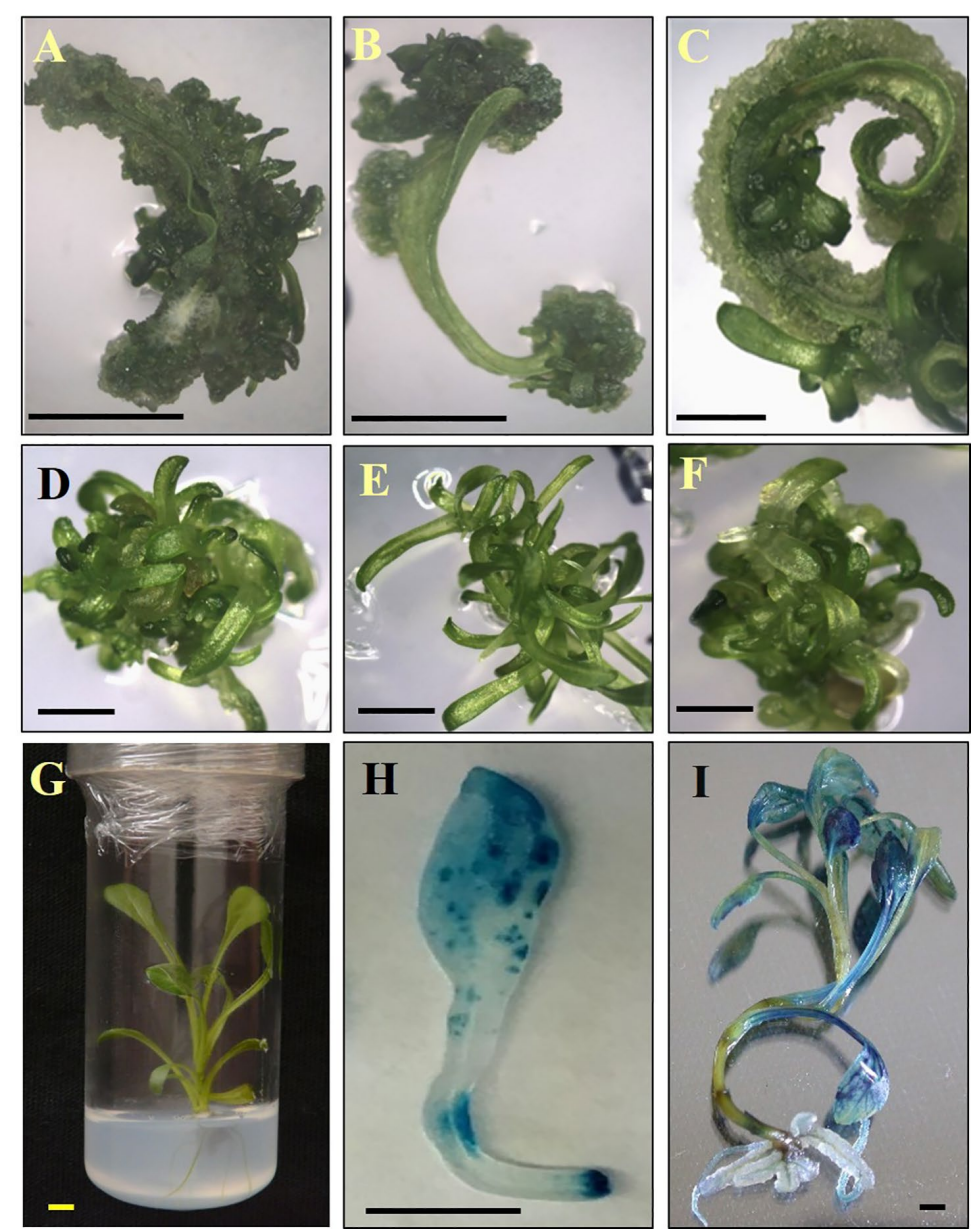
Regeneration and transformation of *Solidago*. **(A–C)** Regeneration of adventitious shoots on leaf explants after 20 days in culture. **(D–F)** Adventitious shoots on elongation media after 60 days in culture. **(G)** Plantlet on rooting medium after 150 days. **(H)** Chimeric GUS-expression pattern in cv. Golden Glory leaf explant inoculated with pKIWI105-35S:uidA. **(I)** Uniform GUS expression in cv. Golden Glory plant transformed with AGLO/pCGN 7001-35S:uidA. A, D, G—cv. Tara; B, E—cv. Ivory Glory; C, F—cv. Golden Glory. Bar = 0.5 cm.

To test the applicability of the regeneration procedure to other *S. canadensis* cultivars, the commercial cvs. TA and GG were selected and adventitious regeneration was evaluated on REM supplemented with zeatin after 35 days of culture. The regeneration efficiency for TA was highest (86%), intermediate for GG (64%), and lowest for IG (40%) (**Figure 1B** and **Table S2**). In TA and GG, adventitious shoots regenerated randomly along the entire explant; in IG, shoots regenerated specifically from the tips (apex and petiole) (**Figures 2A–C**). No other obvious differences were observed during the regeneration process. After 45 days, adventitious shoots were transferred to EM until plantlets formed (**Figures 2D–F**). After ca. 1.5 additional months, plantlets were transferred to rooting medium (**Figure 2G**). Once a well-developed root system was established, plantlets were transferred to the greenhouse where they developed and flowered normally.

### *Agrobacterium*-Mediated Transformation of *Solidago*

**Figure 3:**
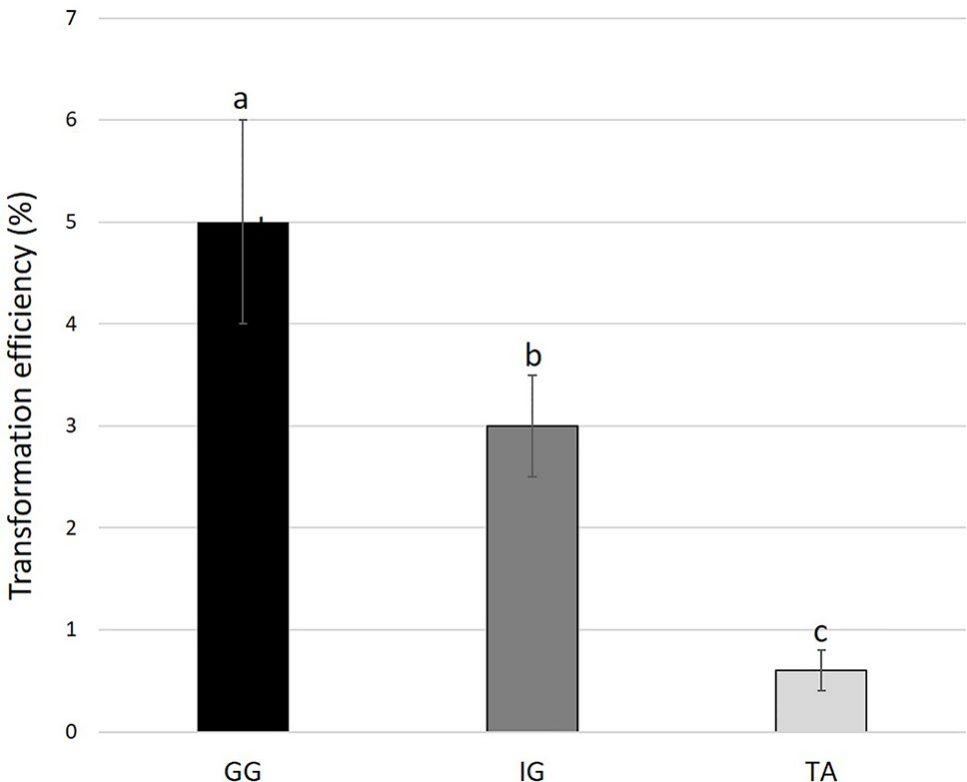
Transformation efficiency of different *Solidago* cultivars. Young leaf explants were cocultivated with AGLO/pCGN7001-35S:GUS-35S. Results are presented as percentage of number of independent GUS-positive plantlets developed on selection media per 100 initial explants. Bars represent mean values of three independent experiments ± SE. Significance of differences between cultivars was calculated using Tukey–Kramer HSD test following one-way ANOVA. Values with different lowercase letters are significantly different (*P* ≤ 0.05). Cultivars: TA, Tara; GG, Golden Glory; IG, Ivory Glory.

**Figure 4:**
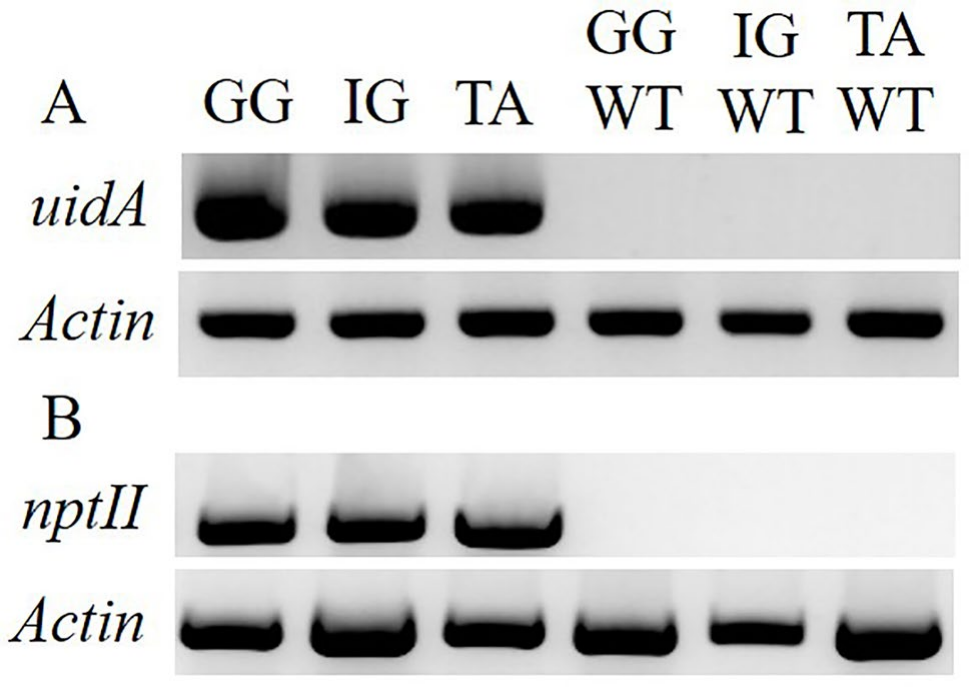
Molecular evaluation of the transgenic nature of *Solidago* plants inoculated with AGLO/pCGN7001-35S:*uidA*. **(A)** RT-PCR analysis of RNA from independent kanamycin-resistant *uidA*-transgenic and control non-transgenic plants. As a reference, PCR amplification of actin was performed. **(B)** PCR analysis of *nptII* and actin in kanamycin-resistant transgenic and control non-transgenic plants. Cultivars: TA, Tara; GG, Golden Glory; IG, Ivory Glory. WT, non-transgenic control plants.

To examine the applicability of agrobacteria to transformation of *Solidago*, leaf explants of the three cultivars were inoculated transiently with *Agrobacterium* strain AGLO carrying the plasmid pKIWI105-35S:uidA. The *uidA* gene in this plasmid encodes GUS, which is not expressed by *Agrobacterium* cells, making it suitable for transient transformation studies (Janssen and Gardner, 1990). Histochemical assay revealed a transformation efficiency (number of GUS-expressing leaf explants out of total number of inoculated explants) of ca. 80% for all cultivars. GUS staining was observed throughout the whole tissue, with strongest expression in the wounded areas of the petiole (**Figure 2H**). To generate transgenic GUS-expressing plants, the explants were inoculated with *Agrobacterium* cells with the pCGN7001 plasmid carrying 35S:uidA and 35S:neomycin phosphotransferase II (nptII). After 3 days of cocultivation, explants were transferred to selective REM for 1 month, during which time adventitious shoots formed. Adventitious shoots were separated from the explants and transferred for ca. 9 months to a selective EM for plantlet development. GG exhibited the highest (5%) transformation efficiency (number of independent GUS-positive plantlets developed per 100 initial explants), ca. 1.7-fold higher than IG (3%) and ca. 8.3-fold higher than TA (0.6%) (**Figure 3**). GUS staining of putative transgenic plantlets revealed uniform GUS expression throughout all of the tissues (**Figure 2I**). To verify the expression of *uidA* in transgenic *Solidago* plants, RT-PCR analysis was performed. Plants expressing *uidA* yielded a DNA fragment of the expected size, whereas no product was detected in the wild-type plants (**Figure 4A**). To further validate the transgenic nature of the independent kanamycin-resistant GUS-expressing plants, PCR analysis of *nptII* was performed. The analysis revealed DNA fragments of the expected size (0.8 kb) in the GUS-expressing plants and none in the controls (**Figure 4B**).

### Stable Transformation of *Solidago* With *Ros1/Del* and *PAP1*

**Figure 5:**
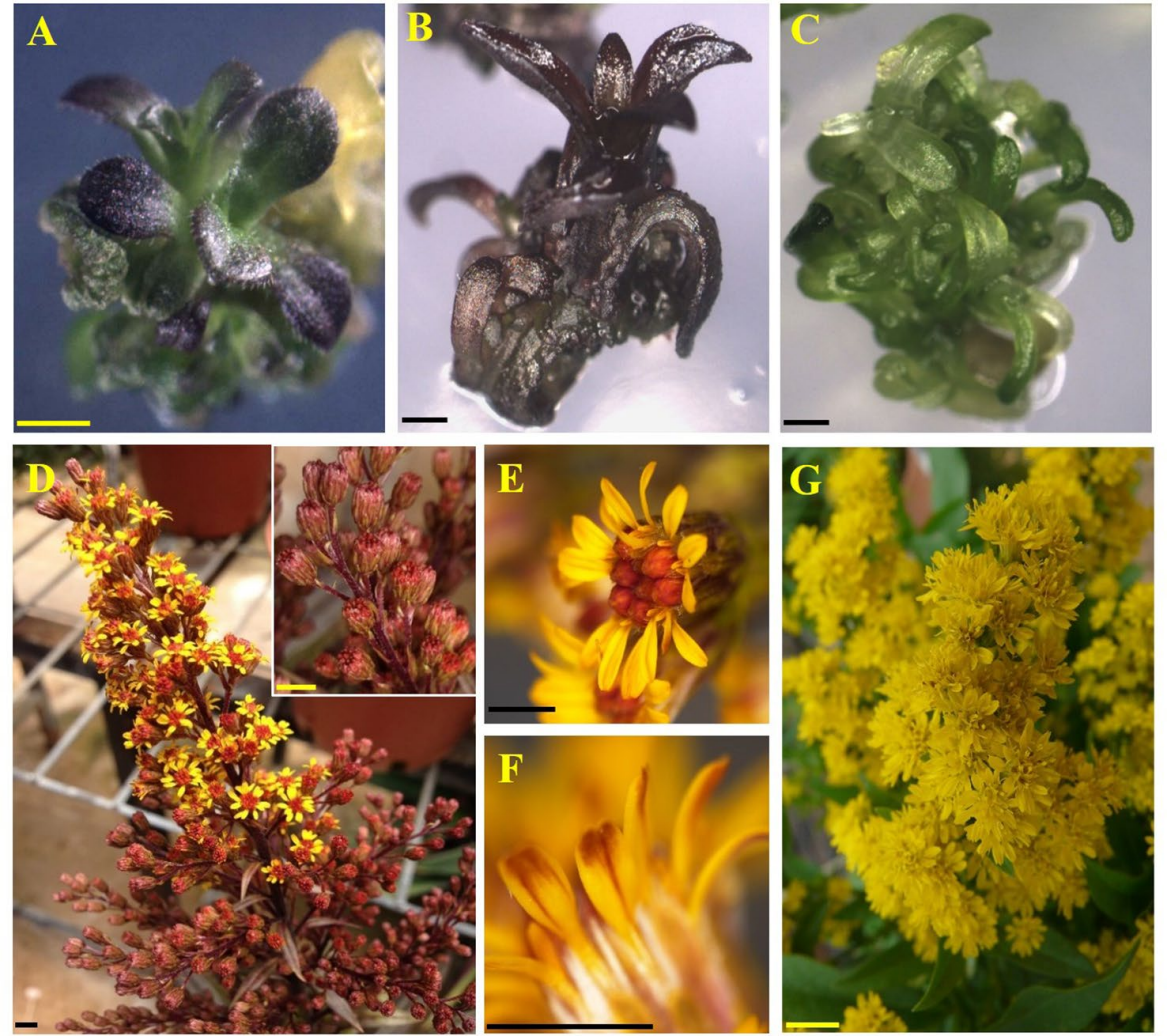
Regeneration of *Solidago* cv. Golden Glory plants accumulating pigments. **(A)** Two-month-old adventitious shoot expressing *Del* and *Ros1*. **(B)** Two-month-old adventitious shoot expressing *PAP1*. **(C)** Two-month-old shoot expressing GUS. **(D)** Inflorescence of line 10. Inset: enlarged view of line 10 closed buds. **(E)** Line 10 flower. **(F)** Abaxial side of line 10 ray floret. **(G)** Inflorescence of transgenic GUS-expressing Golden Glory. Bar = 0.1 cm.

**Figure 6:**
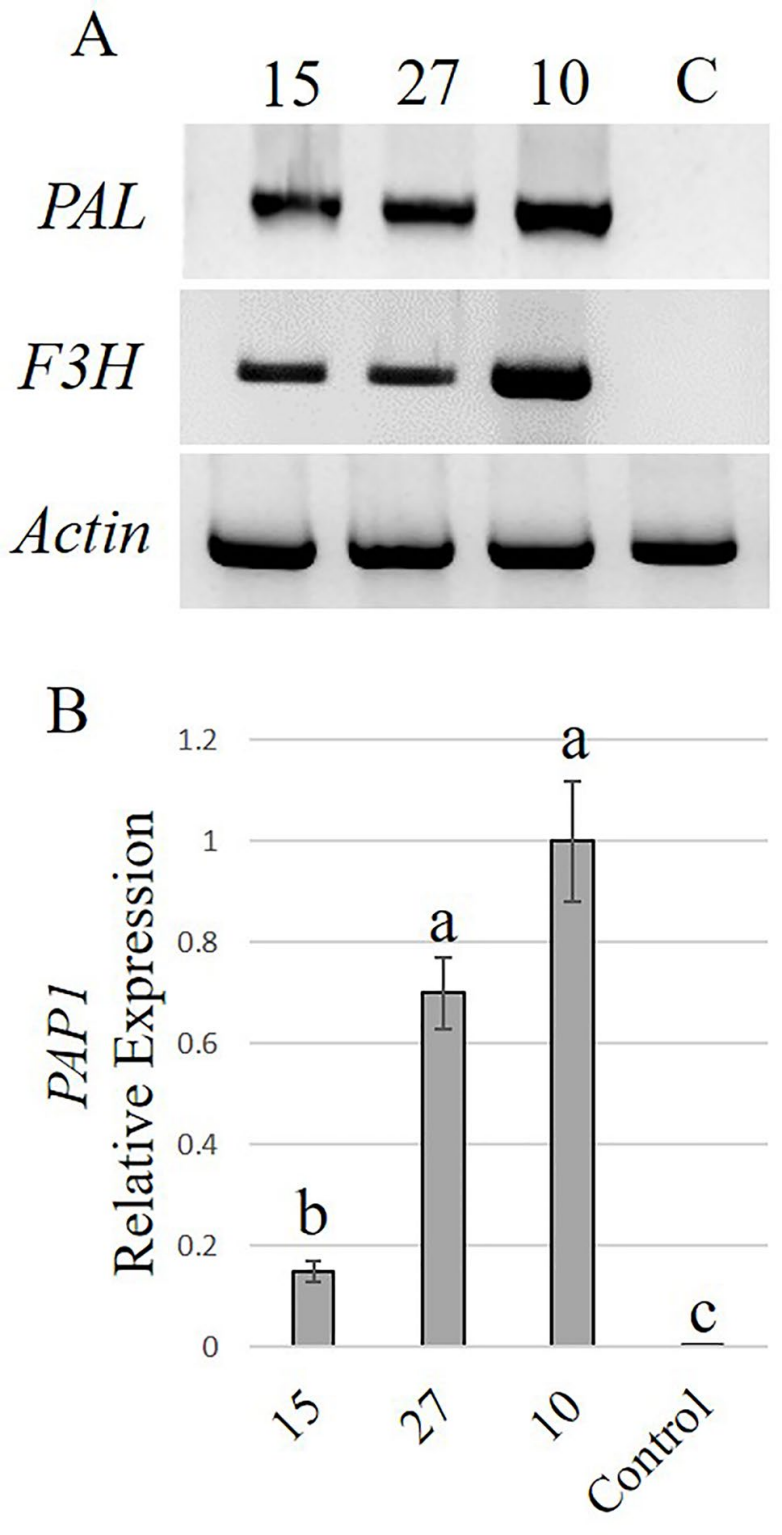
Activation of the phenylpropanoid pathway in *Solidago* flowers expressing *PAP1*. **(A)** RT-PCR analysis of phenylalanine ammonia-lyase (PAL) and flavanone 3-hydroxylase (F3H) transcripts in independent *PAP1*-transgenic plants and control kanamycin-resistant *uidA*-transgenic plants. As a reference, PCR amplification of actin was performed. **(B)** Quantitative real-time PCR analysis (qPCR) of *PAP1* transcript levels in flowers of independent *PAP1-*transgenic lines. Data were normalized to *actin*. Data represent means of three independent experiments ± SE. Significance of differences was calculated using Tukey–Kramer HSD test following one-way ANOVA. Values with different letters are significantly different (*P* ≤ 0.05). 10, 15, 27—Lines 10, 15, 27, respectively; C, control.

**Figure 7:**
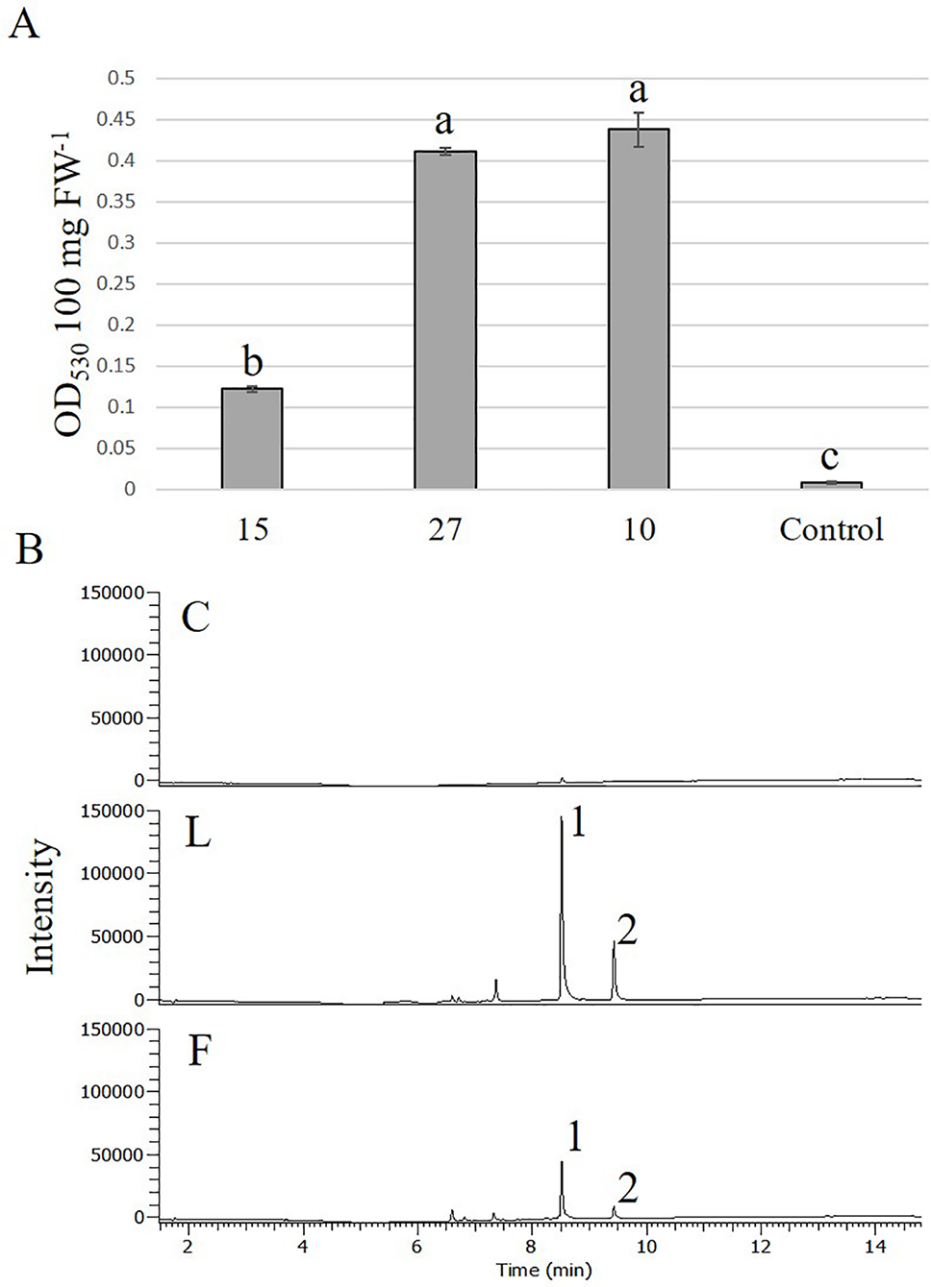
Accumulation of anthocyanins in *Solidago* flowers expressing *PAP1*. **(A)** Anthocyanin accumulation in flowers of different transgenic *PAP1*-expressing *Solidago* plants. Anthocyanins were extracted with acidic methanol and measured using a spectrophotometer. Data represent means of three independent experiments ± SE. Significance of differences was calculated using Tukey–Kramer HSD test following one-way ANOVA. Values with different letters are significantly different (*P* ≤ 0.05). 10, 15, 27—Lines 10, 15, 27, respectively; C, control; FW, fresh weight. **(B)** LC–MS analysis of anthocyanidins accumulating in leaves (L) and flowers (F) of PAP1-transgenic line 10 and control plants (C). 1, delphinidin; 2, methylated delphinidin.

With the highest transformation efficiency, cv. GG was selected for *Agrobacterium*-mediated transformation. In an attempt to generate transgenic *Solidago* plants that accumulate anthocyanins in their flowers, a newly constructed binary vector carrying *Ros1* and *Del* driven by the 35S promoter and 35S-driven *PAP1* (Ben Zvi et al., 2012) were used. To validate the ability of the *Ros1*/*Del* vector to promote accumulation of anthocyanins, tobacco plants were transformed with this construct. Transgenic plants showed a pinkish pigmentation phenotype in both vegetative and reproductive tissues, which was not present in non-transgenic plants, confirming the vector’s functionality (**Figures S1A, B**). RT-PCR analyses of the transgenic tobacco validated the expression of *Ros1* and *Del* in the transgenic tissues (**Figure S1C**). GG explants were transformed with either *Ros1*/*Del* or *PAP1*. Distinct purple pigment accumulation was observed in the leaves of adventitious shoots regenerated from *Ros1*/*Del*-transformed explants, whereas shoots of *PAP1*-transformed plants were uniformly pigmented (**Figures 5A, B**). No pigmentation was observed in shoots regenerated from the control *uid:A*-transformed explants (**Figure 5C**). Several lines of 35S:*Ros1*/*Del* and 35S:*PAP1* plants were transferred to the greenhouse and all of them flowered normally, with no apparent physiological differences from control plants. As 35S:*Ros1*/*Del* plants matured, the leaves’ pigmentation faded and flowers of 35S:*Ros1*/*Del* lines were true-to-type yellow-colored (**Figure S2A**)and not distinguishable from control flowers (**Figure 5G**). RT-PCR analysis of *Ros1* and *Del* validated the expression of both genes in the flowers of the transgenic plant lines, despite the lack of visible pigmentation (**Figure S2B**). In contrast, several independent 35S:*PAP1* plants maintained pigmentation at various levels in the leaves and stems throughout development and showed red-pigmented flowers; whereas the disc florets were uniformly red, the ray florets were mainly yellow, with red pigments concentrated at the periphery of the abaxial side and in streaks at the center on both sides (**Figures 5D–F** and **S3A, B**). To confirm the expression of *PAP1* in independent 35S:*PAP1* lines 10, 15, and 27, RNA was extracted from flowers and subjected to RT-PCR analysis. *PAP1* expression was detected in transgenic flowers, but not in the control *uidA*-expressing plants (**Figure S3C**). To confirm that PAP1 activates expression of genes of the anthocyanin-biosynthesis pathway, expression of PAL and F3H was analyzed by RT-PCR in 35S:PAP1 as compared to control *uidA*-expressing plants. As neither genomic nor transcriptomic data are currently available, degenerate primers to conserved regions of these genes were used. In the 35S:PAP1 lines, PCR yielded fragments of 986 bp for PAL and 367 bp for F3H, whereas in the *uidA*-expressing control, no PCR products were detected (**Figure 6A**). Sequencing of these products confirmed their authenticity: BLAST analysis of PAL and F3H PCR products against the Asteraceae database revealed the top hits (over 96% identity) to PAL in lettuce (*Lactuca sativa*) and sunflower (*Helianthus annuus*) and to F3H in *Callistephus chinensis* and *Erigeron breviscapus*, respectively (**Figure S4**). To analyze PAP1 transcript levels in 35S:PAP1 flowers of the independent lines, qPCR analysis was performed. Lines 10 and 27 accumulated significantly higher transcript levels than line 15. As expected, no PAP1 transcript was detected in control flowers (**Figure 6B**). Anthocyanin levels in PAP1-transgenic lines also accumulated to their highest levels in lines 10 and 27 (**Figure 7A**). To characterize anthocyanins accumulating in PAP1-transgenic line 10 plants, LC–MS analysis was performed. Delphinidin and its methylated derivative were identified in the leaves and flowers of PAP1-transgenic lines, but not in the control uidA-transgenic plants (**Figures 7B** and **S5A–C**).

## Discussion

Flower pigmentation is a trait of major importance to the ornamental plant industry, which is constantly seeking cultivars with novel colors, making it a main goal for breeders (Azadi et al., 2016; Noda, 2018). Limited gene pools hamper achieving this goal in some species, leaving them with a fixed set of colors, or only one color, as with gypsophila and *Solidago*. In some instances, manipulating the biosynthetic pathways by introducing enzymes or transcription factors, or even a partial pathway, *via* genetic engineering, has proven to be successful (Xie et al., 2006; Katsumoto et al., 2007; Ben Zvi et al., 2012; Chandler and Sanchez, 2012; Brugliera et al., 2013; Nishihara et al., 2013; Polturak et al., 2016). However, a prerequisite for genetic engineering is the development of a repeatable transformation and regeneration protocol. In this study, we developed such a protocol, and obtained novel-colored *Solidago* flowers.

### The Effect of Different Cytokinins on *Solidago* Regeneration

A cytokinin’s ability to promote regeneration in various species has been shown to be dependent on its type, on additional cytokinins/phytohormones in the media, and on explant type and genotype (Zhang et al., 2001; Liu et al., 2010; Haddadi et al., 2013; Masekesa et al., 2016; Armas et al., 2017). In lettuce (*L. sativa*), which like *Solidago*, is a member of the Asteraceae family, cotyledon explants show improved regeneration efficiencies on media supplemented with zeatin or low concentrations of BA (Mohebodini et al., 2011; Armas et al., 2017). In another Asteraceae representative, chrysanthemum (*Chrysanthemum morifolium*), BA was found to be the most efficient cytokinin for direct regeneration (Lu et al., 1990). TDZ was found effective for shoot regeneration in strawberry (*Fragaria × ananassa*) leaf explants, while zeatin produced shoot regeneration from shoot-tip explants (Haddadi et al., 2013). In *Solidago*, the efficiency of adventitious shoot regeneration from leaf explants was also strongly affected by the type of cytokinin supplemented to the REM for the commercial cv. IG. Whereas explants grown on media with TDZ and BA showed similar low efficiencies, zeatin was the most potent cytokinin, yielding ca. 1.5-fold higher regeneration efficiency than the other two. Armas et al. (2017) reported a similar zeatin effect on lettuce cotyledon explants, but the developed shoots had an abnormal morphology. This was not the case in *Solidago*, where all of the regenerated shoots developed normally, with no apparent aberrancies. Zeatin had an even greater effect on the two other *Solidago* cultivars tested, yielding a regeneration efficiency of 86% and 64% for TA and GG, respectively. The regeneration patterns varied among the three cultivars. IG shoots regenerated mainly from the tips of the leaf explant, similar to regeneration described by Li et al. (2012), whereas GG and TA developed shoots from both the tips and middle of the explant. These results indicated that zeatin can promote efficient adventitious shoot regeneration from leaf explants of various *Solidago* cultivars.

### *Agrobacterium*-Mediated Transformation of *Solidago*

Transformation mediated by *A. tumefaciens* has been successfully applied to numerous Asteraceae family members, e.g., sunflower, lettuce, chrysanthemum, and dahlia (Muller et al., 2001; Weber et al., 2003; Mohebodini et al., 2011; Otani et al., 2013; Noda et al., 2017). In the genus *Solidago*, successful transformation has only been reported for *Solidago nemoralis*, using *Agrobacterium rhizogenes* for the generation of hairy root cultures (Gunjan et al., 2013). Hence, *Solidago*’s ability to undergo *A. tumefaciens*-mediated transformation was tested. We transiently inoculated young leaf explants with agrobacteria carrying pKIWI105-35S:*uidA*. GUS staining of *Solidago* explants revealed efficient transient transformation for all three cultivars, manifested by a high percentage (ca. 80%) of explants expressing GUS.

For the generation of stable GUS-transformed *Solidago* plants, binary plasmid pCGN7001 carrying 35S:*uidA* and 35S:*nptII* was harnessed, enabling effective selection of transgenic tissues on kanamaycin. Stable GUS-expressing plants were generated for all three cultivars, demonstrating that the developed system is not variety-specific. The stable transformation efficiencies varied among the cultivars, even though their transient transformation efficiencies were similar. GG was found to be the most competent, with the highest percentage of independent GUS-positive plantlets and a transformation efficiency of ca. 5%. TA showed the lowest stable transformation efficiency, despite the fact that it had the highest regeneration efficiency. These results further emphasized the strong cultivar-dependence of regeneration capacity and cell amenability to transformation (Zuker et al., 1998; Cheng et al., 2004).

### Stable Transformation of *Solidago* With *Ros1*/*Del* and *PAP1*

*Solidago* flowers are monochromatically yellow due to carotenoid accumulation (Horvath et al., 2010). However, previous studies have shown *Solidago’*s ability to produce flavonoids, such as quercetin (Apati et al., 2002; Apati et al., 2006). Thus, in an attempt to generate novel-colored flowers, anthocyanin regulators *Ros1* and *Del* from snapdragon and *PAP1* from *Arabidopsis* were introduced into *Solidago* plants. Only PAP1-expressing transgenic lines accumulated delphinidin and its methylated derivative in flowers and leaves of mature plants. The levels of anthocyanins in the analyzed lines were directly correlated with the expression levels of PAP1, with lines 10 and 27 showing higher expression levels of PAP1 and of anthocyanins than line 15. In petunia, PAP1 has been shown to activate PAL and F3H (Ben Zvi et al., 2008). The activation of PAL and F3H by PAP1 in *Solidago*, as revealed by RT-PCR, confirms induction of the anthocyanin-biosynthesis pathway in PAP1-transgenic lines. The lack of these transcripts in flowers of control plants may explain the lack of anthocyanins in flowers of non-transgenic *Solidago*. Delphinidin and its methylated derivatives were the only anthocyanin aglycones identified by the LC–MS analysis. Direction of the metabolic flux toward the delphinidin branch suggests that in *Solidago*, DFR has a higher affinity to dihydromyricetin, anthocyanidin synthase has a higher affinity to leucodelphinidin, and/or flavonoid 3′-hydroxylase is either non-functional or cannot be induced by PAP1. The lack of genomic/transcriptomic and biochemical data in *Solidago* prevents further detailing of the regulation of metabolic flow in these transgenic plants.

*Ros1*/*Del*-expressing and *PAP1*-expressing transgenic plants showed anthocyanin pigmentation in the vegetative tissues, but only *PAP1* plants developed pigmented flowers. This might be explained by the fact that transcription factors vary in their ability to activate/repress genes, mainly due to promoter characteristics, genetic background, tissue, and species. For example, in snapdragon, MYB proteins Ros1, Ros2, and Venosa, derived from a recent gene duplication, induce the expression of different anthocyanin-biosynthesis enzymes (Schwinn et al., 2006). Ros1 and the bHLH transcription factor Del interact to induce late anthocyanin-biosynthesis genes in snapdragon (Schwinn et al., 2006). However, ectopic coexpression of *Ros1* and *Del* in tomato fruit also induces the expression of PAL and chalcone isomerase (Schwinn et al., 2006; Butelli et al., 2008). In several plant systems, anthocyanin regulators MYB and bHLH have been shown to require the formation of an MBW protein complex to exert their effect (Koes et al., 2005; Ramsay and Glover, 2005; Albert et al., 2014; Verweij et al., 2016). In petunia petal limb, MYB AN2, bHLH AN1, and the WD40 AN11 act in concert to regulate anthocyanin biosynthesis (deVetten et al., 1997; Quattrocchio et al., 1999; Spelt et al., 2000; Koes et al., 2005; Albert et al., 2014; Verweij et al., 2016). Loss-of-function mutations in each of the components prevents the formation of the MBW complex, resulting in a white petal phenotype (deVetten et al., 1997; Quattrocchio et al., 1999; Spelt et al., 2000). Interestingly, this complex also activates MYB27 which represses anthocyanin synthesis by converting it into a repression complex and by preventing the complex formation through suppression of *AN1* (Albert et al., 2014). These examples demonstrate the elaborate network of positive and negative regulators involved in anthocyanin production. Since only PAP1 yielded anthocyanin-pigmented transgenic *Solidago* flowers, we suggest that PAP1, as compared to Ros1/Del, is more compatible for binding the cofactors and activating target promoters in *Solidago*, or is less prone to negative regulation, or a combination of these. These suggestions would also explain the differences in pigmentation patterns of *PAP1*-transgenic flowers, which demonstrated a contrast between the highly pigmented ray florets and the disc florets that accumulated anthocyanins mainly in the tip of the abaxial side, while the adaxial side remained yellow. Alternatively, post-transcriptional gene silencing may be responsible for the color patterning, as found for petunia cvs. Night Sky, Picotee and Star, where small interfering RNA accumulates in the white sections and spatially represses the biosynthetic enzyme chalcone synthase, resulting in a bicolored phenotype (Koseki et al., 2005; Saito et al., 2006; Morita and Hoshino, 2018). Future genomic/transcriptomic studies integrated with analyses of metabolic shunts in transgenic tissues should provide new insights into the machinery regulating phenylpropanoid biosynthesis in *Solidago*.

Here we present a novel genetic-transformation protocol that allows advanced molecular breeding techniques to overcome classical breeding limitations for the generation of *Solidago* plants with new traits. In the future, these plants may constitute a platform for the development of novel color mixes, based on anthocyanin, carotenoid and betalain pigments.

## Data Availability Statement

All datasets generated for this study are included in the article/**Supplementary Material**.

## Author Contributions

All authors performed the experimental work, discussed the results, and commented on the article. OS and JR wrote the article.

## Funding

This work was supported by the Israel Science Foundation (grant 2511/16).

## Conflict of Interest

The authors declare that the research was conducted in the absence of any commercial or financial relationships that could be construed as a potential conflict of interest.
