# Supplementary material for: Ectopic Expression of PAP1 Leads to Anthocyanin Accumulation and Novel Floral Color in Genetically Engineered Goldenrod (Solidago canadensis L.)
Source: Front Plant Sci. 2019 Nov 27;10:1561. doi: 10.3389/fpls.2019.01561 (PMC6890609; doi:10.3389/fpls.2019.01561)
Supplement: Supplementary file 1 [file DataSheet_1.pdf]

Cytokinin type	EFS (%)	AE
**BA**		
Experiment 1	23	1.11
Experiment 2	27	1.04
Experiment 3	28	1
Average	**26±1.2**	**1.05±0.02**
**Zeatin**		
Experiment 1	34	1.15
Experiment 2	30	1.33
Experiment 3	38	1
Average	**34±1.8**	**1.16±0.1**
**TDZ**		
Experiment 1	19	1.13
Experiment 2	21	1.2
Experiment 3	23	1.1
Average	**21±1**	**1.14±0.02**

Supplemental Table S1. Percentage of explants forming shoots (EFS) and average number of shoots per explant (AE) produced from of *Solidago* cv. Ivory Glory on media containing BA, zeatin or TDZ.

Cultivar	EFS (%)	AE
**Ivory Glory**		
Experiment 1	36	1.1
Experiment 2	41	1.1
Experiment 3	31	1.1
Average	**36±3**	**1.1±0.0**
**Golden Glory**		
Experiment 1	55	1.2
Experiment 2	68	1.1
Experiment 3	41	1.3
Average	**54±8**	**1.2±0.04**
**Tara**		
Experiment 1	64	1.4
Experiment 2	58	1.3
Experiment 3	62	1.5
Average	**61±2**	**1.14±0.02**

Supplemental Table S2. Percentage of explants forming shoots (EFS) and average number of shoots per explant (AE) produced from *Solidago* cultivars Ivory Glory, Golden Glory and Tara. Leaf explants were cultured on media containing 0.1 mg L^{-1} NAA supplemented with 1.5 mg L^{-1} zeatin.

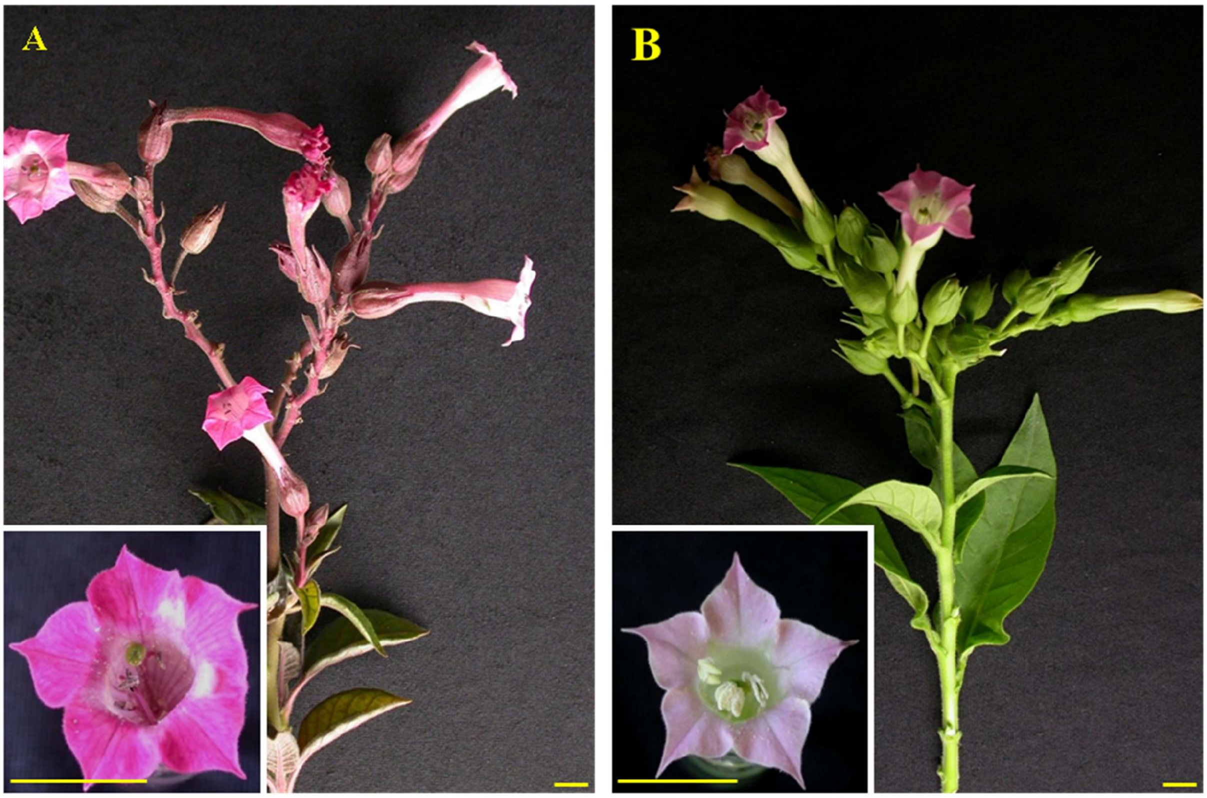

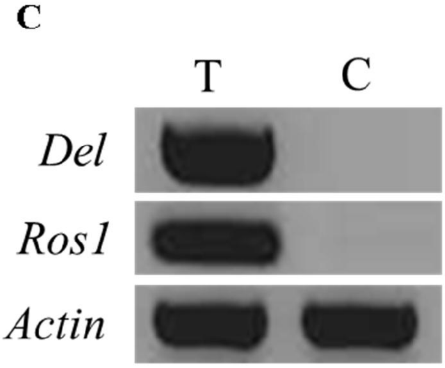

Fig. S1 (A) Transgenic *Del*/*Ros1*-expressing tobacco. (B) Wild-type tobacco. (C) RT-PCR analyses of RNA from transgenic tobacco expressing *Del*/*Ros1* (T) and control non-transgenic plant (C). As a reference, PCR amplification of actin was performed. Bar = 1 cm.

A

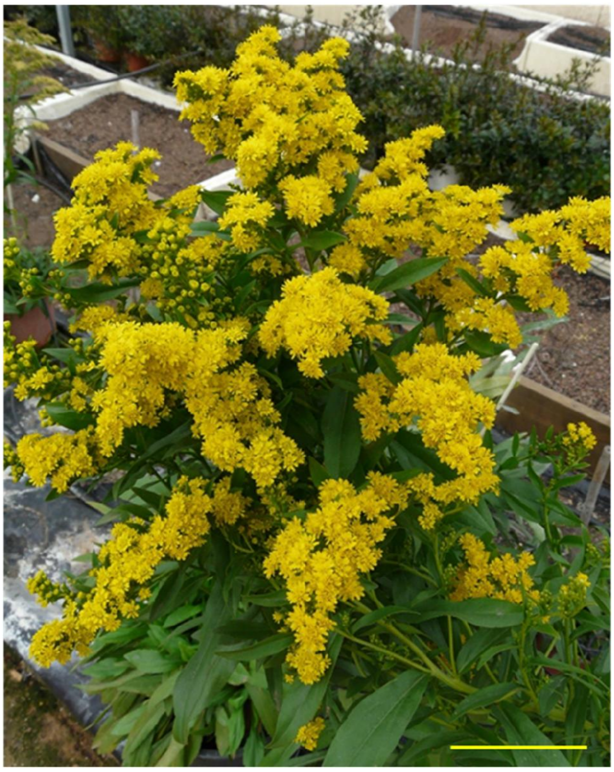

B

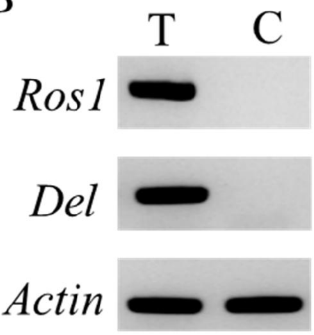

Fig. S2 (A) Transgenic *Del*/*Ros1*-expressing *Solidago* cv. Golden Glory (GG). (B) RT-PCR analyses of RNA extracted from flowers of transgenic *Del*/*Ros1*-expressing GG (T) and control GUS-expressing transgenic GG (C). As a reference, PCR amplification of actin was performed. Bar = 10 cm.

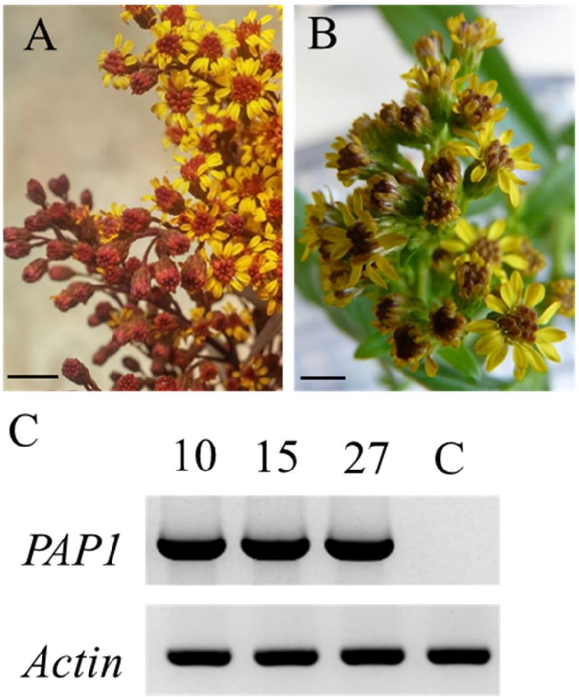

Fig. S3 Inflorescences of transgenic *PAP1*-expressing *Solidago* cv. Golden Glory (GG) line-27 (A) and line-15 (B). (C) Molecular evaluation of the transgenic nature of *Solidago* cv. Golden Glory plants expressing *PAP1*. RT-PCR analysis of RNA from independent kanamycin-resistant *PAP1*-transgenic plants and control kanamycin-resistant *uidA*-transgenic plants. As a reference, PCR amplification of actin was performed. Bar = 1 cm.

Phenylalanine ammonia-lyase (PAL)

TGGCCGATCCAGCTCCCGGCCGCCTGGCGGCCGCGGGAATTCGATTTTGGTGCTACTTCTCA
GCGGAGGACCAAGAATGGTGGAGCTCTTCAAAAGGAACTTATTAGGTTTTTGAATGCTGGA
ATCTTTGGCAACGGCACCGAATCAAGCCACACACTCCCACATTCAGCAACTAGAGCTGCCA
TGCTAGTAAGAATCAACACCCTTCTCCAGGGTTACTCTGGCATCCGTTTCGAGATTTTGGAA
GCCATCACCAAGTTCCTCAACACCAATGTCACCCCTTGCTTGCCTCTTCGTGGGACCATTAC
TGCTTCTGGAGATCTTGTACCGTTGTCCTACATTGCTGGACTTCTTACCGGCCGCCCCAATTC
CAAAGCCCTTGGTCCCAACGGTGAAATCCTCAATGCCGAAAAGGCATTCAAGCTAGCCGGT
GTGGAAGGTGGGTTCTTTGAGCTACAGCCCAAAGAAGGTCTAGCACTTGTTAATGGTACAG
CTGTGGGATCTGGCATGGCCTCTATGGTTCTTTTTGAAGCCAATGTACTTGCATTGTTATCA
GAAGTCTTGTCTGCTATTTTCTCTGAAGTTATGCAAGGAAAGCCCGAGTTTACTGACCACCT
GACACACAAACTGAAGCATCATCCAGGCCAGATTGAAGCTGCAGCAATTATGGAGTACATT
TTAGATGGAAGTGATTATGTAAAGGCTGCACAAAAGGTCCACGAAATGGACCCTTTACAAA
AACCTAAACAAGACCGATATGCTCTAAGAACTTCACCTCAATGGCTCGGTCCACTAATTGA
GGTCATCCGGTCATCAACTAAAATGATCGAAAGAGAGATCAACTCCGTGAACGATAGCCCT
TTGATTGATGTCTCAAGAAACAAGGCCTTACATGGTGGTAACTTCCAAGGACACCAATTGG
TGTTTCCATGGATAACACACGTCTGGCTATTGCTGCAATAGGAAAACTCATGTTTGCTCAAT
TC

Flavanone 3-hydroxylase (F3H)

GGCGGCCTGATAAGCCCAATGAATGGAGGGCTGTTACTGAAGAATATAGCAAGGTGTTAAT
GGATCTCGCTTGCAAGCTTCTAGAGGTGTTATCCGAGGCCATGGGGCTTGAGAAAGAAGCC
CTTACAAAGGCATGTGTTGATATGGACCAAAAGGTAGTGGTGAATTACTACCCAAAATGTC
CTCAGCCCGACCTTACATTGGGCCTGAAACGACATACTGATCCAGGAACGATCACATTGTT
GCTTCAGGACCAAGTTGGCGGGCTTCAAGCGACTCGTGATGGTGGCAAGAGTTGGATCACG
GTTAAGCCGGTTGAAGGTGCTTTCGTGGTTAATCTTGGCGATCATGGACATTATTTGAGCAA

Fig. S4 Sequenced fragments amplified using degenerate primers for *Solidago phenylalanine ammonia-lyase* (*PAL*) and *flavanone 3-hydroxylase* (*F3H*).

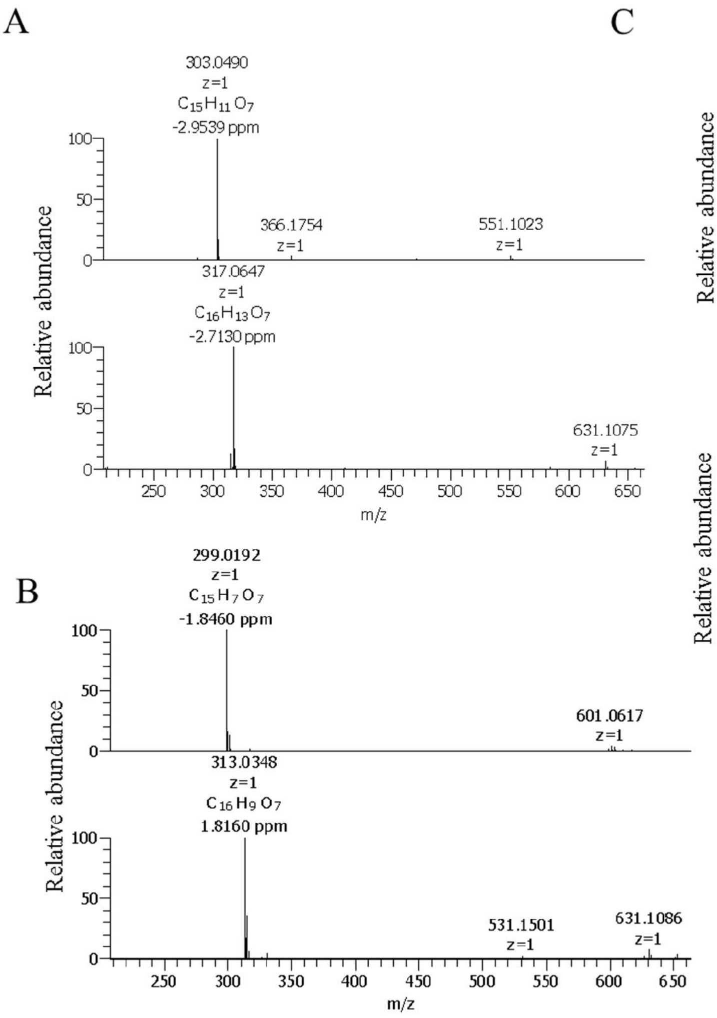

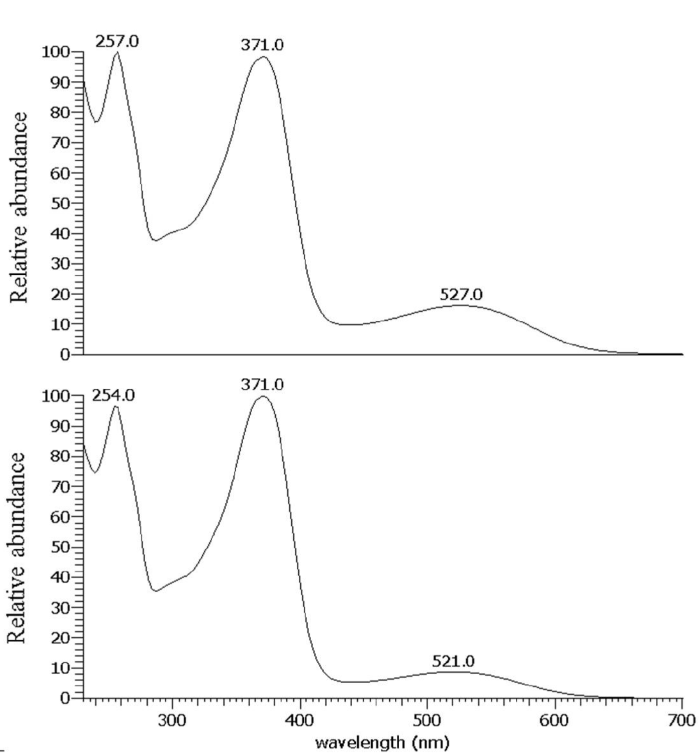

Fig. S5 (A) Positive ESI mass spectra recorded for a compound eluted at RT=8.5 min (upper, m/z 303.0490 (C15H11O7) atomic composition is consistent with that of delphinidin) and at RT 9.4 min (lower, m/z 317.0647 (C16H13O7) atomic composition is consistent with methylated delphinidin). (B) Negative ESI mass spectra recorded for a compound eluted at RT=8.5 min (upper, m/z 299.0192 (C15H7O7) atomic composition is consistent with that of delphinidin) and at RT 9.4 min (lower, m/z 313.0348 (C16H9O7) atomic composition is consistent with the methylated delphinidin). (C) UV spectrum of compounds eluted at RT 8.5 min (delphinidin, upper) and at RT 9.4 min (methylated delphinidin, lower).
